# Phosphatidylglycerol Supplementation Alters Mitochondrial Morphology and Cardiolipin Composition

**DOI:** 10.3390/membranes12040383

**Published:** 2022-03-31

**Authors:** I Chu, Ying-Chih Chen, Ruo-Yun Lai, Jui-Fen Chan, Ya-Hui Lee, Maria Balazova, Yuan-Hao Howard Hsu

**Affiliations:** 1 Department of Chemistry, Tunghai University, Taichung 40704, Taiwan; joyce1024joyce1024@gmail.com (I.C.); michaelchen8900@gmail.com (Y.-C.C.); iz51034@yahoo.com.tw (R.-Y.L.); rivabox@hotmail.com (J.-F.C.); elankisa007@gmail.com (Y.-H.L.); 2 Institute of Animal Biochemistry and Genetics, Centre of Biosciences, Slovak Academy of Sciences, 840 05 Bratislava, Slovakia; maria.balazova@savba.sk

**Keywords:** phosphatidylglycerol, cardiolipin, tafazzin, mass spectrometry

## Abstract

The pathogenic variant of the *TAZ* gene is directly associated with Barth syndrome. Because tafazzin in the mitochondria is responsible for cardiolipin (CL) remodeling, all molecules related to the metabolism of CL can affect or be affected by *TAZ* mutation. In this study, we intend to recover the distortion of the mitochondrial lipid composition, especially CL, for Barth syndrome treatment. The genetically edited *TAZ* knockout HAP1 cells were demonstrated to be a suitable cellular model, where CL desaturation occurred and monolyso-CL (MLCL) was accumulated. From the species analysis by mass spectrometry, phosphatidylethanolamine showed changed species content after *TAZ* knockout. *TAZ* knockout also caused genetic down-regulation of *PGS* gene and up-regulation of *PNPLA8* gene, which may decrease the biosynthesis of CLs and increase the hydrolysis product MLCL. Supplemented phosphatidylglycerol(18:1)_2_ (PG(18:1)_2_) was successfully biosynthesized to mature symmetrical CL and drastically decrease the concentration of MLCL to recover the morphology of mitochondria and the cristae shape of inner mitochondria. Newly synthesized mature CL may induce the down-regulation of *PLA2G6* and *PNPLA8* genes to potentially decrease MLCL production. The excess supplemented PG was further metabolized into phosphatidylcholine and phosphatidylethanolamine.

## 1. Introduction

Barth syndrome is caused by the pathogenic variant of the *TAZ* gene on the X chromosome [1,2,3,4]. The dysfunction of tafazzin enzyme disrupts cardiolipin (CL) remodeling (a process for acquiring suitable acyl moieties for CL), which causes the accumulation of monolyso-CL (MLCL) and a quantity decrease in the mature CL [5,6,7,8]. The imbalance of CL and MLCL decreases the function of mitochondria [9]. Due to the link between Barth syndrome and CL remodeling, MLCL/CL has become a diagnosis index for Barth syndrome [10,11].

CL is a glyceride molecule with two phosphatidylglycerol (PG) moieties. In eukaryotic cells, CL usually contains four long fatty acyl chains with 16–22 carbon atoms [12,13,14,15,16]. The double bonds in these fatty acyl moieties of CL are critical for cone shape formation to maintain the curvature of the inner mitochondrial membrane [17,18,19]. The mature CLs in mammals and eukaryotes, such as CL(18:1)_4_ and CL(18:2)_4_ [20,21], are mainly symmetrical. The presence of symmetrical CLs in the structure of the mitochondrial membrane is assumed to be suitable for achieving the full mitochondrial function [20]. Several pieces of evidence have indicated that CL participates in the synthesis of the mitochondrial inner membrane [22]; stabilization of electron transport chain complexes II, III, and IV [5]; ATP synthesis [23]; mitophagy [24]; and apoptosis [25].

Unlike most phospholipids synthesized in endoplasmic reticulum, CL is uniquely biosynthesized in the mitochondria of eukaryotes [21]. A CL synthase combines cytidine diphosphate diacylglycerol (CDP-DAG) and PG to *de novo* synthesize a nascent CL [9,26]. In the remodeling process in a *Drosophila* model of Barth syndrome, the fatty acyl moiety of the nascent CL can be hydrolyzed using calcium-independent phospholipase A2 beta (iPLA_2_β) or iPLA_2_γ to produce MLCL [27]. The nascent CL is then remodeled by tafazzin into mature CL on the mitochondrial membrane shown in Saccharomyces cerevisiae [28]. MLCL can be reacylated to CL by using lysocardiolipin acyltransferase (ALCAT1) or MLCL acyltransferase (MLCLAT1) [29,30,31]. Because of the exchange of acyl moieties, most phospholipids directly or indirectly participate in the metabolic pathway of CL. Tafazzin can bind with phospholipids; hydrolyze phosphatidylcholine (PC), phosphatidylethanolamine (PE), and CL substrate to lysoPC, lysoPE, and MLCL, respectively; and transfer the acyl chain to target MLCL [4,32,33]. Remodeling is not specific to any particular fatty acyl chain; however, it has been identified to favor unsaturated 18-carbon fatty acids in the mitochondria of Spodoptera frugiperda 9 (Sf9) insect cell [4].

CL metabolism is closely related to other phospholipids. The dysfunction of tafazzin in Barth syndrome patients can increase the PE content in mitochondria and decrease the CL content [8]. PE and CL are both related to the curvature of the phospholipid membrane model in vitro [34]. When mitochondrial PE is 20–30% depleted, cell growth and mitochondria activity are inhibited in PSB-2 cells, mutant CHO cells [35]. The elimination of PE production induced through phosphatidylserine decarboxylase gene knockout changes the morphology of mitochondria [36,37]. PE has been shown to have an overlapping function with CL in mitochondria. Similar to CL, PE can interact with ETC complex to stabilize the complex structure [38,39,40,41]. When mitochondria lack CL, PE may compensate for CL deficiency to maintain the mitochondrial function [42,43,44]. Moreover, PC is the most abundant phospholipid in cells and mitochondria and the most crucial substrate for CL remodeling enzymes [4,32,33]. The phospholipid transfer protein StAR related lipid transfer domain containing 7 (Stard7) is responsible for the specific transport of PC to mitochondria, and the intra-mitochondrial lipid transfer [45,46]. Stard7 deficiency disrupts the transfer of PC to mitochondria, which results in the alteration of the structure of mitochondria and homeostasis of epithelial cells [47]. The PC species is considerably changed in patients with Barth syndrome [8].

The dysfunction of mitochondria caused by the *TAZ* knockout could be restored by adeno-associated virus (AAV)-mediated gene replacement therapy in a mouse model [48], indicating that the damage of mitochondria is reversible. An in vitro experiment has shown that PG is a precursor of mitochondrial CL and a functional substitute of CL for stabilizing electron transport complexes [49]. PG deficiency decreases oxidative phosphorylation in the mitochondria of saccharomyces cerevisiae cells [50]. Supplemented PG has been shown to incorporate into CL, and it changes the CL profile in macrophage-like RAW 264.7 cells [51]. In this study, we investigated PG supplementation effects on the CL-deficient cellular model of Barth syndrome through the comprehensive lipidomic analysis of glycerol phospholipids.

## 2. Material and Methods

### 2.1. Materials

*TAZ* knockout of HAP1 (Δ*TAZ*) by clustered regularly interspaced short palindromic repeats (CRISPR)/CRISPR-associated protein 9 (Cas9) is a customized cell line purchased from Horizon Discovery (Cambridge, UK). Dulbecco’s modified eagle medium (DMEM) and fetal bovine serine (FBS) were purchased from Invitrogen (St. Louis, MO, USA). Bradford protein binding assay, iScript complementary DNA (cDNA) synthesis kit and SYBR Green system Super Mix were purchased from BioRad (Montreal, QC, Canada). Tetra-myristoyl cardiolipin CL(14:0)_4_ and phosphatidylglycerol(18:1)_2_ were purchased from Avanti Polar Lipids (Alabaster, AL, USA). Anti-DDK antibody was purchased from Novus (Littleton, CO, USA). Formic acid was purchased from Sigma-Aldrich (St. Louis, MO, USA). Acetonitrile and isopropanol were purchased from JT Baker (Phillipsburg, NJ, USA). Acclaim RSLC 120 C18 column was purchased from Thermo (Ottawa, ON, Canada).

### 2.2. Megabase-Scale CRISPR/Cas9 Knockout of TAZ Gene

The *TAZ* gene knockout of HAP1 cell (Δ*TAZ*) was a customized service from Horizon discovery. Briefly, *TAZ* gene was edited by Megabase-scale CRISPR/Cas9 (Clustered regularly interspaced short palindromic repeats/CRISPR-associated proteins). The guide RNA and donor DNA were designed and constructed, and then the HAP1 cells were transfected. Single cell clone were selected and the modification were confirmed. The genetic edited cell line were expanded. Two nucleotides were removed to cause the frame shift, resulting in a premature stop codon and a 66-amino acid fragment without enzymatic activity after translation. The mutation results were confirmed by RNA sequencing again.

### 2.3. Phosphatidylglycerol (18:1)_2_ Supplementation

HAP1 ΔTAZ was cultured in the medium of 90% DMEM, 10% Fetal bovine serum (FBS), 0.5% Penicillin-Streptomycin (Pen-Strep) and 5% CO_2_ at 37 °C. In a 6-cm plate, 4 × 10^5^ cells were plated with 4 mL medium. In the PG supplementation experiments, 6.4 μL of 25 mg/mL PG(18:1)_2_ in ethanol was supplemented twice at 0 and 24 h to reach a final concentration of 50 μM. The cells were harvested and counted at 48 h after initial PG supplementation.

### 2.4. TEM Observation of Mitochondria in HAP1 and ΔTAZ

The cover glass was placed in a 35-mm cell culture plate and the plate was exposed under ultraviolet light for 24 h. A total of 4 × 10^5^ cells were plated into the 35-mm plate and incubated in 5% CO_2_ at 37 °C for 24 h. The glass covers were immersed in 2.5% glutaraldehyde for 1.5 h to fix the cells. After washed with phosphate buffered saline (PBS) at pH 7.0 twice, the glass covers were immersed in 1% Osmium tetroxide for one hour. The sample were dehydrated by ethanol and embedded in spurr resin at 72 °C for 12-h polymerization. The spurr resin were sliced by Leica UC7 slicer and placed in a cupper net. The mitochondria in the fixed cells were observed under Hitachi HT-7700 transmission electron microscopy (TEM) at 100 kV.

### 2.5. Real-Time Quantitative PCR

The collected HAP1 and HAP1ΔTAZ cells were suspended by 1 mL of TRIZOL reagent and then mixed with 200 μL of chloroform. After centrifugation at 12,000 rpm for 15 min at 4 °C, 500 μL of the upper phase was collected and added 500 μL of isopropanol. To collect the precipitate, the samples were kept at −20 °C for 20 min and then placed at room temperature for 10 min. The precipitate was washed with 500 μL of ethanol and centrifuged at 9000 rpm for 5 min at 4 °C. Then, 20 μL of Diethylpyrocarbonate (DEPC) treated water was added and the samples were heated to 50 °C for 5 min. The cDNA synthesis Kit from Bio-Rad, Hercules, CA, USA was utilized for the mRNA reverse transcription. Following the protocol of the kit, 1 μg of the purified mRNA was mixed with 4 μL iscript reaction mix and 1 μL iscript reverse transcriptase for reverse transcription. The sample was heated to 42 °C for 30 min and 85 °C for 5 min. After DNA quantitation, the samples were stored at −20 °C.

To initiate quantitative PCR, we mixed 50 ng of cDNA, 10 pmole of forward/reverse primer and 10 μL SYBR Green Supermix (Bio-Rad) and then added water to a total volume of 20 μL. The sequences of the primers were shown in Supplemental Table S1. The mixtures were subjected to a MiniOpticon Real-Time PCR System. The heat cycling for PCR were 180 s at 95 °C for initial polymerase activation, 40 cycles of 15 s at 95 °C and 90 s at 60 °C. At the end of the polymerase chain reaction (PCR), the temperature was increased 0.1 °C per second to acquire the melting curve.

### 2.6. Lipid Extraction

The total lipid extraction followed the Bligh-Dyer’s method as our previous experiments [15]. The internal standards, 250 ng of CL(14:0)_4_, 250 ng of MLCL(18:2)_3_, 250 ng of PS(14:0)_2_, 125 ng of PG(14:0)_2_, 125 ng of PE(14:0)_2_, 125 ng of PC(14:0)_2_ were added to the cell pallets along with 2 mL of methanol/1% formic acid. The collected cells were sonicated at 80% amplitude of 125 watt for 20 s on ice for 3 times, added 1 mL of dichloromethane and further vortexed for 10 min. Then, 1 mL of dichloromethane and 1 mL of distilled deionized water were mixed with samples and vortexed for another 10 min. The lower organic phase was collected and stored at −20 °C.

### 2.7. Mass Spectrometry Analysis

The lipid extracts were dried by nitrogen gas, and re-dissolved with 400 µL of acetonitrile/2-propanol (1:9) with 0.1% formic acid and 10 mM ammonium formate. The sample vials were stored in −20 °C until the analysis by Bruker LC/MS Ion-Trap. Reverse phase HPLC gradient was operated in an Acclaim RSLC 120 C18 column at a flow rate of 0.2 mL/min at 55 °C. The gradient contained solution A: acetonitrile:water (60:40), 10 mM ammonium formate, 0.1% formic acid, and solution B: isopropanol: acetonitrile (90:10), 10 mM ammonium formate, 0.1% formic acid. The acquired mass spectrometry (MS) data were further analyzed by Bruker DataAnalysis (ver.4.1). The extract ion current (XIC) of each phospholipid species was quantitated by their relativity of XIC to internal standard. Each type of total phospholipid is the sum of all quantitated species of its type. The percentage of each phospholipid was the ratio of XIC of each phospholipid species to total XIC of its type.

### 2.8. Statistical Analysis

Standard deviations were calculated by the standard deviation (STDEV) function in Microsoft Excel for the error bars of the histograms in the figures and student’s *t*-tests were applied to all triplicated data.

## 3. Results

### 3.1. Genetic TAZ Knockout Effects in the Barth Syndrome Cellular Model

**Figure 1:**
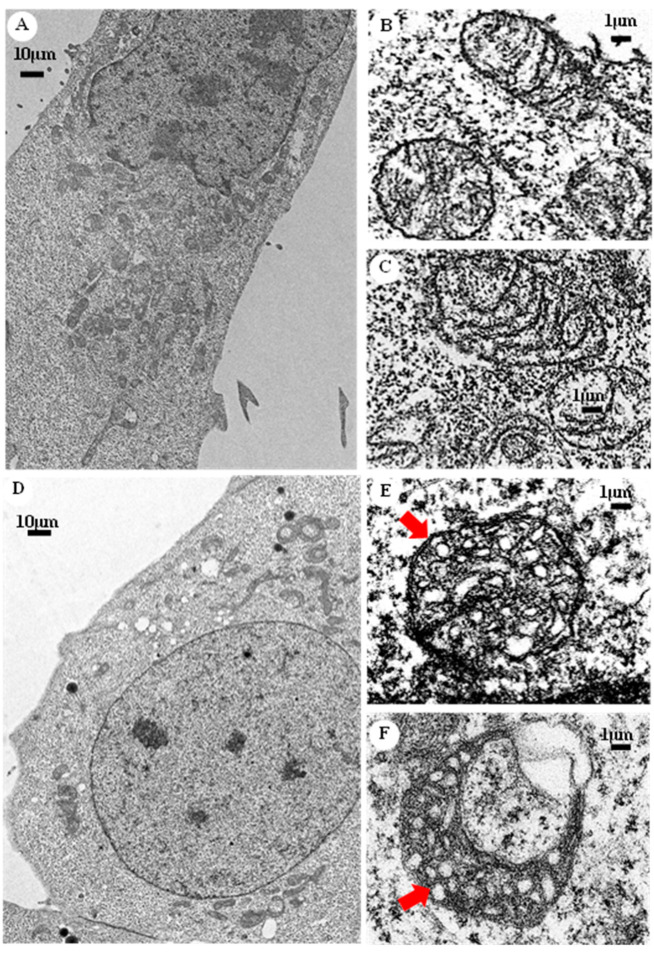
Mitochondrial morphology of HAP1WT cells and HAP1Δ*TAZ* cells. HAP1 WT cells were fixed and embedded in resin and healthy mitochondria could be observed by TEM under 1000× (**A**) and 10,000× (**B**,**C**). After genetic editing of the *TAZ* gene by CRISPER-Cas9, the curved mitochondria with internal bubble morphology in HAP1Δ*TAZ* cells were observed by TEM under 1000× (**D**) and 10,000× (**E**,**F**).

We generated a cellular model of Barth syndrome by using the genetic *TAZ* knockout of the HAP1 cell line, which was confirmed using RNA sequencing. The deletion of two bases caused a frame shift and resulted in a short translated fragment containing 66 amino acids of tafazzin protein. The morphology of the mitochondria was further observed using a transmission electron microscope (Hitachi HT-7700) (Figure 1). Under 1000× magnification, circular-shaped healthy mitochondria were observed around the nucleus in HAP1 cells (Figure 1A). The zoomed-in view of 10,000× magnification illustrated the regular and smooth layers of the inner mitochondrial membrane (Figure 1B,C). In the HAP1ΔTAZ cell, the mitochondria became narrow, extended, and circular and scattered in the complete cell (Figure 1D). The inner mitochondrial membrane formed bubbles and irregular curves (Figure 1E,F).

### 3.2. TAZ Knockout Caused Phospholipid Changes

**Figure 2:**
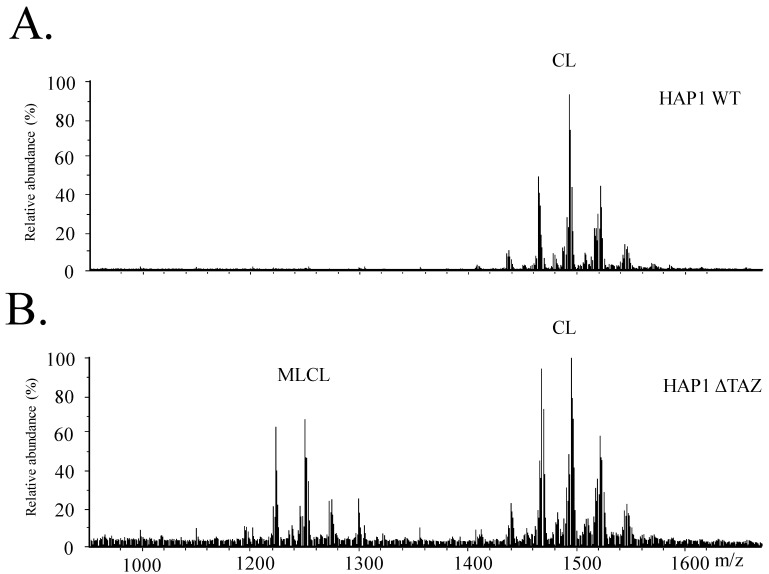
The mass spectrum of CL and MLCL of HAP1 and HAP1ΔTAZ. Total lipids were extracted from the HAP1 and HAP1ΔTAZ cells by Bligh Dyer’s method and analyzed by LC/MS. The mass spectrum of HAP1 (**A**) and HAP1ΔTAZ (**B**) were shown the drastic increase of MLCL after TAZ knockout.

We further evaluated the effects of MLCL accumulation on Δ*TAZ* by using ion-trap mass spectrometry (Figure 2). Under the reverse phase chromatography, MLCL was eluted earlier than CL, showing the high hydrophobicity of CL. Both MLCL and CL were ionized as negative ions. The intensity of the MLCL mass spectra was relatively lower than the intensity of CL in the HAP1 wild type (WT) samples. *TAZ* knockout drastically increased the concentration of MLCL and ratio of MLCL/CL The relative abundance of MLCL was drastically increased from 0.3 fmole/cell in HAP1 to 1.7 fmole/cell in Δ*TAZ*.

**Figure 3:**
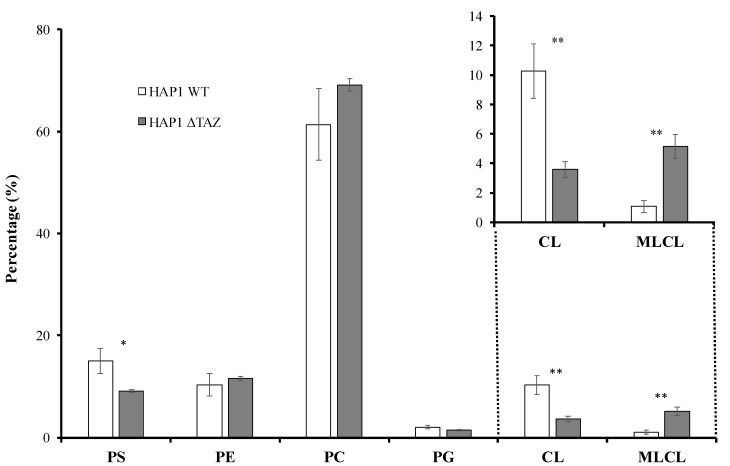
The phospholipid ratio in the HAP1 and HAP1ΔTAZ cells. The total phospholipids were extracted from the HAP1 and HAP1ΔTAZ cells by Bligh and Dyer method. The phospholipid quantities were quantified by liquid chromatography (LC)/MS. The up-right panel is the zoom in of CL and MLCL, showing the significant decrease of CL and increase of MLCL in HAP1ΔTAZ cells. The experiments were done in triplicate, and the error bars are the standard deviation of the triplicates. The standard deviation and the *t*-test (* *p* < 0.05, ** *p* < 0.01) are calculated by the function of Microsoft Excel. The phosphatidylserine (PS), phosphatidylethanolamine (PE), phosphatidylcholine (PC), phosphatidylglycerol (PG), cardiolipin (CL) and monolysocardiolipin (MLCL) were quantified.

The intensity of the mass spectrum may not represent the accurate MLCL/CL ratio. Therefore, the accurate quantitation of the MLCL and CL were calculated by the sum of the area of each MLCL and CL species. The genetic knockout of *TAZ* caused a 65% decrease in CL and a 5.3-fold accumulation of MLCL (Figure 2 and Figure 3). The ratio of MLCL/CL increased from 0.10 to 1.4. Besides the changes of CL and MLCL, the percentage of PS concentration had a significant 38.9% decrease after *TAZ* knockout. The percentage changes of PC, PE and PG concentration were not significant after student’s *t*-test.

### 3.3. CL Species Changes upon TAZ Knockout

**Figure 4:**
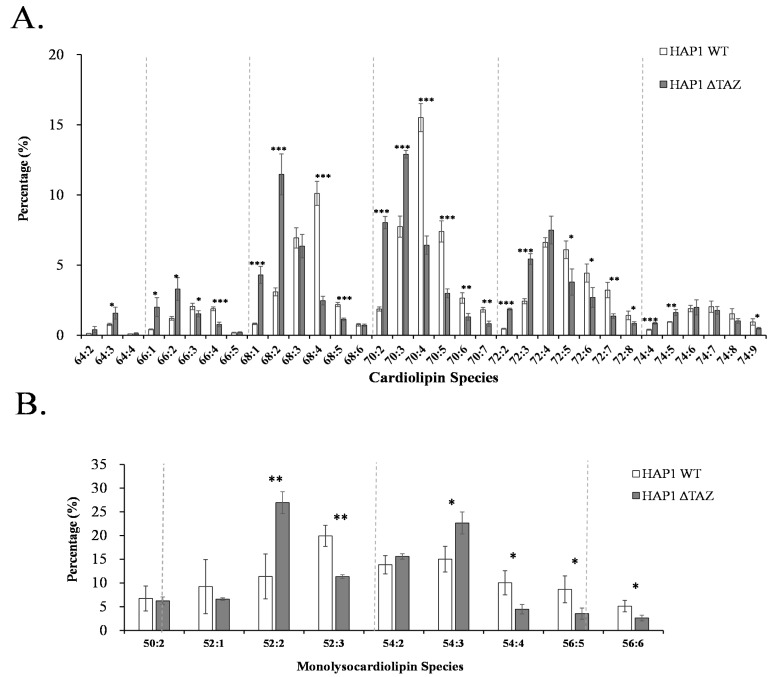
Relative percentages of CL and MLCL in HAP1WT and HAP1ΔTAZ cells. HAP1WT and HAP1ΔTAZ cells were harvested. There were 33 CL (**A**) and 9 MLCL (**B**) species analyzed by IonTrap mass spectrometry. TAZ knockout drastically changed the profile of CL and MLCL. The data were triplicated and statistically analyzed by Microsoft Excel *t*-test (* *p* < 0.05, ** *p* < 0.01, *** *p* < 0.001).

The genetic knockout of *TAZ* caused a decrease in the double bond number in all five CL groups, namely CL(66), CL(68), CL(70), CL(72), and CL(74) (Figure 4A). The numbers in parentheses indicate the total number of carbon atoms in the acyl moieties of phospholipid. Because the mature CL usually contains unsaturated fatty acyl chains, the elevation in saturated fatty acyl moieties in CL explained the disruption of CL remodeling of the unsaturated fatty acyl chain onto the nascent CL. *TAZ* knockout effects on the saturation of the MLCL carried the same trends as of the CL (Figure 4B). MLCL contains three acyl moieties. The percentage increases of MLCL(54:3) and MLCL(52:2) indicated the formation of the MLCL species, containing multiple 18:1 fatty acid moieties.

### 3.4. PG/PC/PE/PS Species Changes after TAZ Knockout

**Figure 5:**
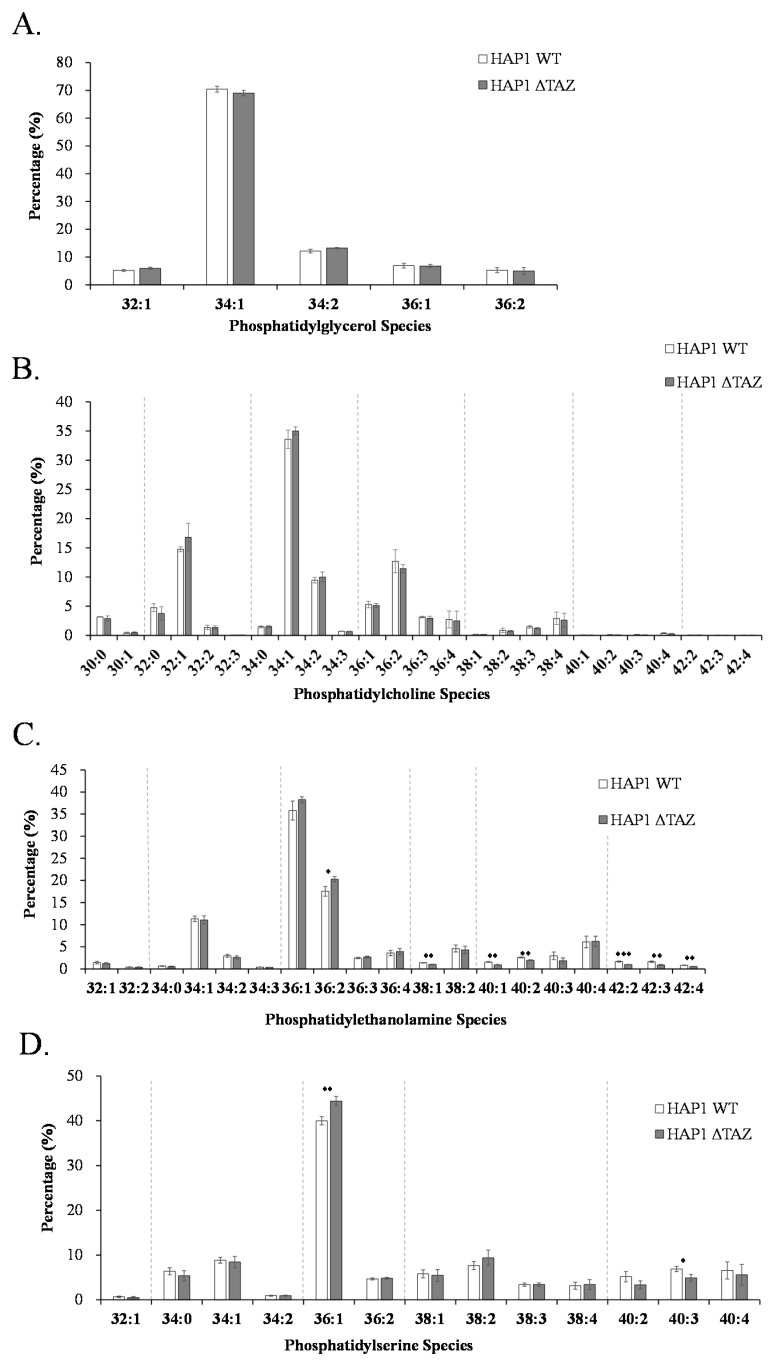
Relative percentages of PG, PC, PE, and PS phospholipids in HAP1 and HAP1ΔTAZ cells. HAP1 and HAP1ΔTAZ cells were harvested. Phospholipids were quantified by mass spectrometry. The 5 species of PG (**A**) and 25 species of PC (**B**) did not have any significant change. Seven out of the identified 19 species of PE (**C**) have significant changes in concentration. The 13 species of PS (**D**) only showed slight changes after quantified by IonTrap mass spectrometry. The data were triplicated and statistically analyzed by Microsoft Excel *t*-test (* *p* < 0.05, ** *p* < 0.01, *** *p* < 0.001).

*TAZ* knockout did not affect the quantity and species of PG (Figure 3 and Figure 5A). Because PG was not disturbed at all, the dysfunction of CL remodeling seem not affect CL synthesis from PG. Because of the continuous production of the nascent CL and the lack of remodeling, cells accumulated the nascent CL. Otherwise, we would have observed the inhibition of CL synthesis, which would have caused PG accumulation.

PC is a potential substrate of tafazzin for CL remodeling. After *TAZ* knockout, we observed marginal increase of PC concentration (Figure 3), but the percentage of each PC species maintained the similar level (Figure 5B). This finding strongly suggested that tafazzin did not used PC as a direct substrate for CL remodeling. Because the remodeling is specific to 18:1 or 18:2, PC species would change after CL remodeling. According to the species changes in CL, we expected the 18:1 acyl chain of PC(18:1)(18:1) to be the moiety transferred through tafazzin. PE is also proposed to be another possible substrate of tafazzin. Although we did not observed significant quantity changes, the acyl chains of PE changed significantly, which indicated that the cellular PE was related to *TAZ* knockout (Figure 5C). The total concentration of PS decreased in Δ*TAZ* cells significantly when using Student’s *t* test. The species of PS did not change, but PS(36:1) had an significant 11% increase (Figure 5D).

### 3.5. Gene Expression of CL Synthesis and Remodeling-Related Genes

**Figure 6:**
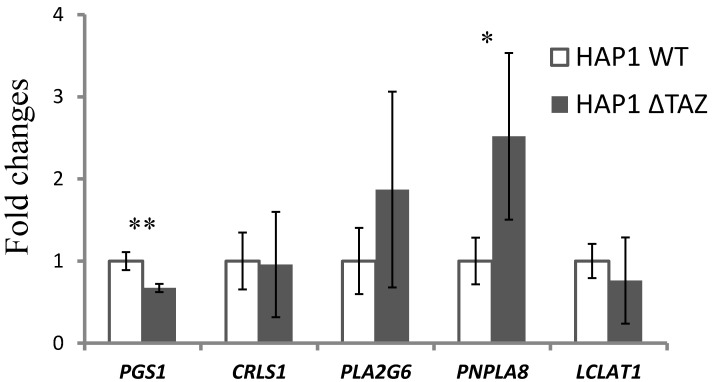
Regulation of the lipid-metabolism related genes in Δ*TAZ* cells. The total RNA of the harvested HAP1 and Δ*TAZ* cells were extracted and then reverse-transcribed to cDNA. The expression levels of CL synthesis: *PGS1* and *CRLS1*, and CL remodeling: *LCLAT1*, *PLA2G6*, *PNPLA8* were quantitated by quantitative reverse transcription PCR (RT-qPCR). The data were triplicated and statistically analyzed by Microsoft Excel *t*-test (* *p* < 0.05, ** *p* < 0.01). *PGS1* and *PNPLA8* showed significant changes after TAZ knockout.

We used RT-qPCR to determine whether the changes in phospholipids were gene-regulated (Figure 6). In HAP1ΔTAZ cells, *PGS1* exhibited 32% (2^−6.95^/2^−6.38^) down-regulation. The unchanged species and concentration of PG indicated that the nascent CL synthesis from PG was not affected by *TAZ* knockout; however, the PG synthesis and CL synthesis from PG likely decreased simultaneously and therefore attained equilibrium.

Because of tafazzin knockout, the cells may have slowed down the production of the nascent CL through the down-regulation of *PGS1*, but not of *CRLS1*. The alternative remodeling pathway may have been activated. The gene expression of the mitochondrial phospholipase *PNPLA8* (iPLA_2_γ) was elevated 2.5 folds (2^−1.27^/2^−2.57^) to hydrolyze the nascent CL. However, the *LCLAT1* gene responsible for transferring the new fatty acyl chain was not significantly up-regulated, which led to the accumulation of MLCL.

### 3.6. PG(18:1)_2_ Supplementation Effect

**Figure 7:**
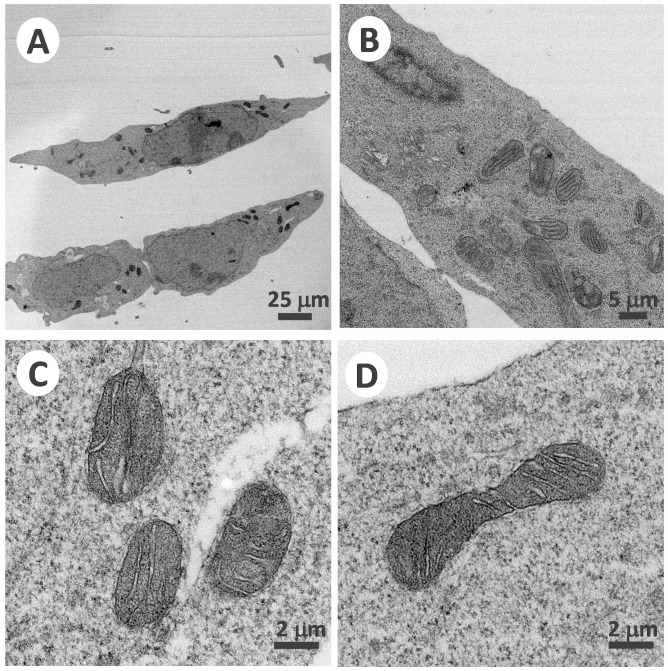
Mitochondrial morphology of HAP1Δ*TAZ* cells after PG supplementation. After PG supplementation, HAP1Δ*TAZ* cells were fixed and embedded in resin. The healthy morphology of mitochondria was observed by TEM under 1200× (**A**), 5000× (**B**) and 20,000× (**C**,**D**).

**Figure 8:**
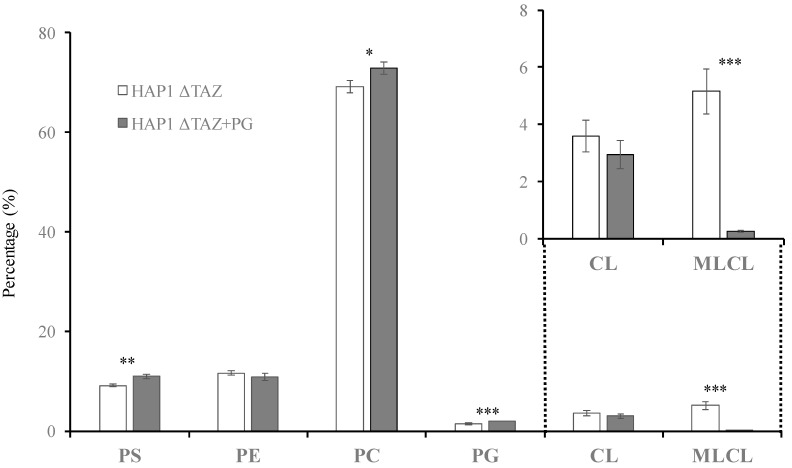
PG(18:1)_2_ supplementation effects on the phospholipids in HAP1ΔTAZ cells. PG(18:1)_2_ was supplemented to the HAP1ΔTAZ cells. The total phospholipids were then extracted by Bligh and Dyer method. The phospholipid quantities were quantified by LC/MS. The up-right panel is the zoom in of CL and MLCL, showing the drastic decrease of MLCL after PG supplementation. The experiments were done in triplicate, and the error bars are the standard deviation of the triplicates. The standard deviation and the *t*-test (* *p* < 0.05, ** *p* < 0.01, *** *p* < 0.001) are calculated by the function of Microsoft Excel.

PG and cytidine diphosphate diacylglycerol (CDP-DAG) are the direct reactants used for CL biosynthesis. It has been shown that CDP-DAG supplementation could not change the CL species and that PG(18:2)_2_ supplementation was not as effective as PG(18:1)_2_ to incorporate into CL [51]_._ Therefore, PG(18:1)_2_ was supplemented to Δ*TAZ* cells. The morphology of the mitochondria in the cells were observed by TEM (Figure 7) and phospholipid was analyzed using mass spectrometry (Figure 8). To our surprise that, we observed the recovery of the mitochondrial morphology after treatment. Although the CL concentration decreased marginally, the MLCL concentration decreased 97%, which caused the MLCL/CL ratio to decrease from 1.4 to 0.085. Notably, PG(18:1)_2_ supplementation elevates 38% of the percentage of PG concentration, which indicated that the PG supplementation successfully incorporated into cells and altered the MLCL/CL ratio. PS elevated 20.3%. The two major phospholipids, PC and PE, have minimal percentage changes. PC merely elevated 5.5% and PE decreased 6.0%, which were the normal compositions of healthy HAP1.

### 3.7. PG(18:1)_2_ Supplementation Effects on CL and MLCL

**Figure 9:**
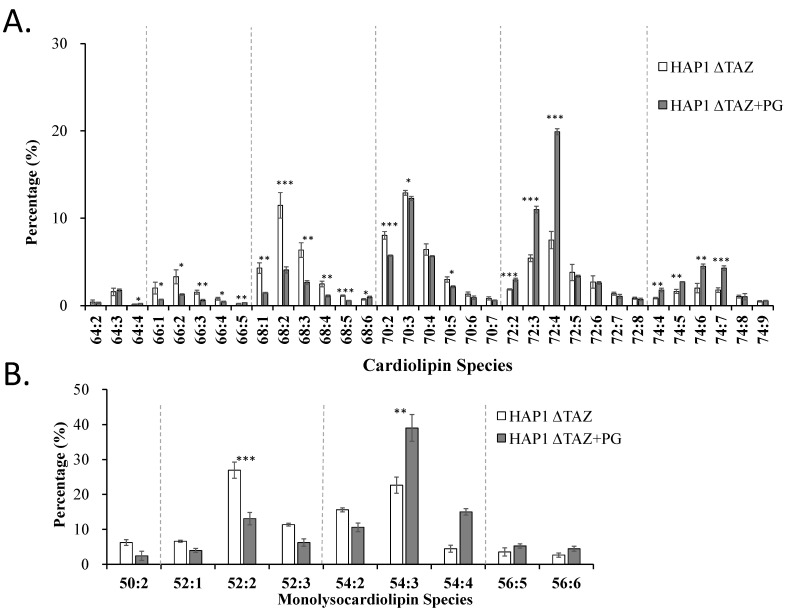
PG(18:1)_2_ supplementation effects on the CL and MLCL profiles in HAP1ΔTAZ cells. HAP1ΔTAZ cells were harvested at 48 h after PG(18:1)_2_ supplementation. The 50 CL species (**A**) and 17 MLCL species (**B**) were analyzed by IonTrap mass spectrometry, showing the elevation of the chain length of both CL and MLCL. The data were triplicated and statistically analyzed by Microsoft Excel *t*-test (* *p* < 0.05, ** *p* < 0.01, *** *p* < 0.001).

PG supplementation was used to decrease the MLCL concentration and MLCL/CL index. We further examined the species changes in CL and MLCL (Figure 9). The results indicated that the short chains CL(66) and CL(68) decreased, whereas the long chains CL(72) and CL(74) increased, which indicated an increase in the fatty acyl chain length. The species CL(72:4) was a symmetrical form of CL(18:1)_4_, which contained the same fatty acyl chains as the supplemented PG(18:1)_2_. The results proved the incorporation of 18:1 fatty acyl chain into CL. Although the overall CL concentration did not increase, the CL species was shifted to a high-18:1 profile.

After PG(18:1)_2_ supplementation, MLCL(54) and MLCL(56) increased, whereas MLCL(50) and MLCL(52) decreased, which indicated an increase in the MLCL chain length. From the pattern of all MLCL groups, MLCL species shifted toward unsaturation with many double bonds. Because of the high concentration of the 18:1 moieties after PG supplementation, MLCL was assumed to carry 18:1 fatty acyl chains. Indeed, we observed the percentage change in MLCL(54:3) and the actual MLCL(54:3) concentration in the cell increased substantially. This result indicated that MLCL was a result of the degradation of excess CL and that the hydrolysis of the acyl chain was selective. The 16:0, 16:1, and 18:0 fatty acyl moieties of MLCL were released, and the 18:1 and 18:2 moieties were preserved. Therefore, our results proved that supplemented PG(18:1)_2_ can not only enhance symmetrical CL synthesis and recover the MLCL/CL ratio but also stimulate the degradation of MLCL and selectively recycle the 18:1 and 18:2 moieties.

### 3.8. PG(18:1)_2_ Supplementation Effects on PG, PC, PE, and PS

**Figure 10:**
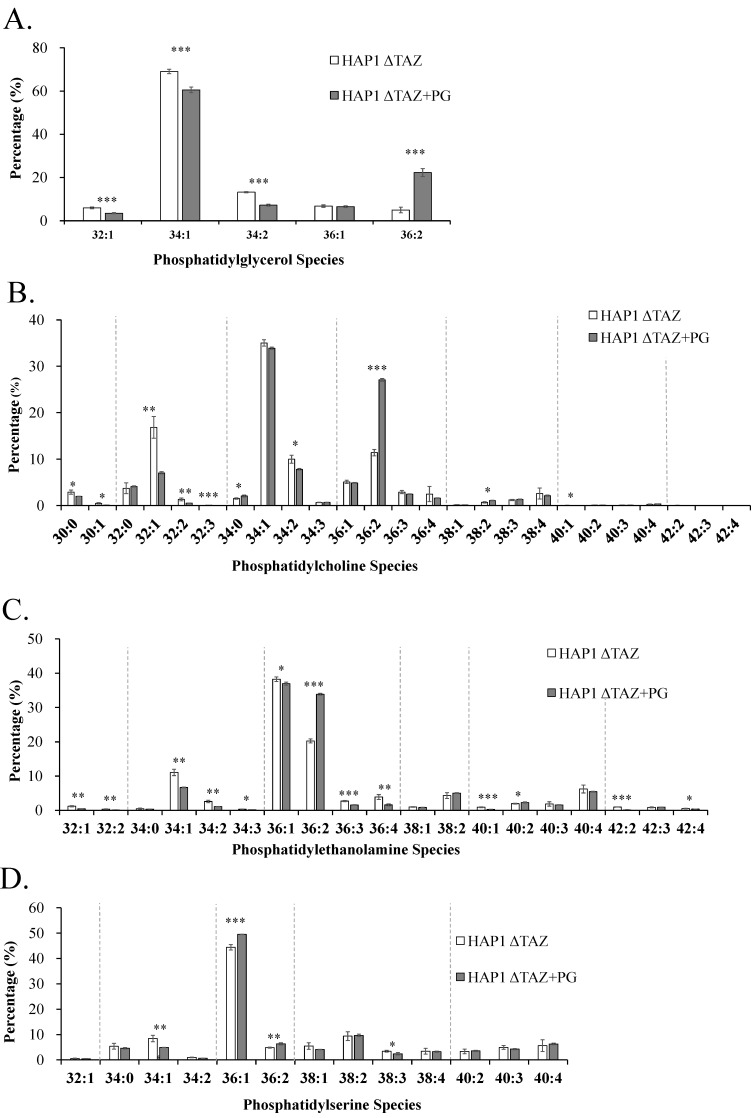
PG(18:1)_2_ supplementation effects on the PG, PC, PE and PS profiles in HAP1ΔTAZ cells. HAP1ΔTAZ cells were harvested at 48 h after PG(18:1)_2_ supplementation. The 5 species of PG (**A**), 25 species of PC (**B**), 19 species of PE (**C**), and 13 species of PS (**D**) were analyzed by IonTrap mass spectrometry. The elevation of 36:2 phospholipid species, containing 18:1 fatty acyl chains appeared in all four phospholipids. The data were triplicated and statistically analyzed by Microsoft Excel *t*-test (* *p* < 0.05, ** *p* < 0.01, *** *p* < 0.001).

We observed an increase in PG(36:2) and decreases in PG(32:1), PG(34:1) and PG(34:2), which indicated the incorporation of PG(18:1)_2_ into cells (Figure 10A). The 18:1 moiety substituted the 16:0 and 16:1 moieties in PG. However, the quantity of the supplemented PG(36:2) should contribute a considerably higher increase than the current results. Because PG is not the main energy source for β-oxidation, the supplemented PG must be further metabolized. The supplemented PG was likely transformed into other types of phospholipids.

Although total PC percentage in the cell was not affected by PG supplementation, the concentration of each PC species was changed (Figure 10B). The PC profile suggests that the amount of the PC(36:2) species substantially increased, which indicates that PG(18:1)_2_ was metabolized into PC(18:1)_2_. The level of decrease in PC(32:1) was close to the level of increase in PC(36:2), and other species did not contribute to a high level of change. These observations indicated that the 18:1 moiety on PG was transacylated to replace the 16:0 moiety and convert it to PC(34:2). The 16:1 moiety of PC(34:2) can be replaced by 18:1 to form PC(36:2).

PG supplementation changed many PE species with 34 and 36 carbons. Among them, PE(36:2) exhibited the highest increases and PE(34:1) exhibited the highest decrease in percentage of concentration (Figure 10C). The pattern indicated that the PE remodeling utilized the acyl chains of PG as a source and that the 18:1 moiety was more favorable than 16:0 acyl moiety. The overall trends of changes are similar between PC and PE. PS only exhibited marginal percentage increases of PS(36:1) and PS(36:2), and decrease of PS(34:1) after PG supplementation (Figure 10D).

### 3.9. Gene Expression of Mitochondrial Phospholipases

**Figure 11:**
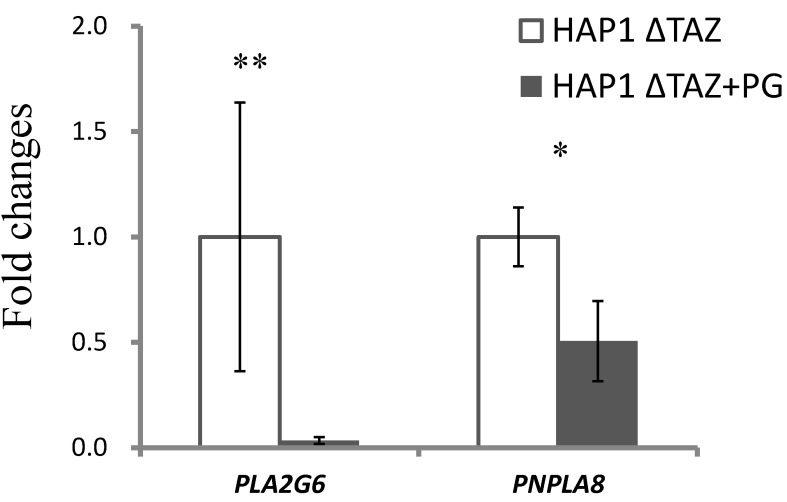
Gene expression of mitochondrial phospholipases after PG supplementation. After PG(18:1)_2_ supplementation, the total RNA of the harvested HAP1ΔTAZ cells were extracted and then reverse-transcribed to cDNA. The decreasing expression levels of *PLA2G6* (iPLA_2_β) and *PNPLA8* (iPLA_2_γ) were quantitated by RT-qPCR. The data were triplicated and statistically analyzed by Microsoft Excel *t*-test (* *p* < 0.05, ** *p* < 0.01).

The phospholipases iPLA_2_β (gene: *PLA2G6*) and iPLA_2_γ (gene: *PNPLA8*) can localize to mitochondria for initiating CL or phospholipid remodeling and degradation. To investigate MLCL degradation, we examined the gene expression of *PLA2G6* and *PNPLA8* (Figure 11). *PLA2G6* and *PNPLA8* genes were 95.5% (2^−11.48^/2^−6.73^) and 49.5% (2^−2.41^/2^−1.38^) down-regulated, which indicated low-fatty-acyl-chain hydrolysis of CL to MLCL.

## 4. Discussion

### 4.1. Effects of the Genetic Knock out of TAZ on Cardiolipin

*TAZ* gene was knocked out in various cellular and animal models to study Barth syndrome. The haploid HAP1 cell line have the well-developed chromosomes, and thus particularly useful for germ cell research, haploid cell studies, or genetic screening. The human HAP1 model generated using Crisper Cas9 exhibited all the crucial characteristics of Barth syndrome, including the wrinkled and twisted morphology of mitochondria with defective mitochondrial respiration (Figure 1). The tafazzin knockdown zebrafish model has shown the of the abnormal cardiac development and cardiac function failure [52]. The HAP1 model showed decreased level of CL and increased level of MLCL (Figure 2 and Figure 3). The null mutation of *taz1*Δ of a temperature-sensitive yeast mutant showed similar reduction of the CL content [53]. The fatty acyl chains of the CL exhibited significant changes in both yeast and HAP1 models. Besides impaired CL remodeling, the induced pluripotent stem cells derived from Barth syndrome patients exhibited an increased MLCL content as the HAP1 model [54]. *TAZ* null mutation also caused flying problems for drosophila, in which the symmetrical CL(16:1)_2_(18:1)_2_ shifted to asymmetrical CL(16:0)(16:1)(18:1)(18:2) [55,56]. A decrease in symmetrical CL(18:2)_4_ and an increase in MLCL were found in the *TAZ* knock-down mouse model [57]. PG supplementation in our gene-edited model elevated the symmetrical CL(18:1)_4_, which will improve the function of mitochondria (Figure 9A).

After tafazzin lost its function, the CL remodeling process in mitochondria ceased, which resulted in a relatively high percentage of newly synthesized immature CLs [58]. Immature CLs in cardiomyocytes exhibited a short saturated fatty acyl chain with relatively few double bonds. These short-chain and saturated CLs exhibited damaged mitochondrial structure and function in the mouse model. The accumulation of MLCL can be observed from the experimental results in this study, which may be caused by the up-regulation of the iPLA_2_γ gene and potentially promote the hydrolysis of immature CL into MLCL. The cells exhibited decreased CL concentration and the increased MLCL concentration led to an imbalance in the MLCL/CL ratio, which jeopardized the mitochondrial function.

### 4.2. Effects of the Genetic Knock out of TAZ on Phospholipids

The synthesis of phospholipids are inter-connected. After *TAZ* knockout, the total quantity and species contents of CL and other phospholipids will be affected. PG is a crucial upstream material for CL biosynthesis. After *TAZ* knockout, downstream acyl chain remodeling did not change the species and the concentration of PG significantly. However, *TAZ* knockout triggered the down-regulation of PG synthase, which might reduce the PG production rate (Figure 6) and partially contributed to the decrease of CL concentration. A previous study of the cultured skin fibroblasts of patients has shown normal PG and CL biosynthesis rate and suggested the acceleration of CL degradation [6]. Therefore, the minor changes of PG could be foreseen.

PC and PE are two proposed substrates for tafazzin [59]. PS synthase 1 (PSS1) can use PC as substrate to synthesize PS [60], whereas PS synthase 2 (PSS2) can use PE as substrate to synthesize PS [61]. PS synthases were regulated by phosphorylation of PS synthase and the concentration of PS [62,63]. Tafazzin knockout caused the decrease of PS concentration, the increase of PC concentration and no change of PE concentration (Figure 3). This result suggests PC could become an efficient substrate for PS in response to tafazzin knockout. The changes of the fatty acyl moieties of PE were much more significant than PC (Figure 5B,C). PE can be further synthesized with elongated fatty acyl chains by PS decarboxylase (PSD) in mitochondria, leading to the changes of the species profile [64]. A high proportion of PS is available in the mitochondria for PE biosynthesis, and the acyl chains of PS may reflect the acyl chain moieties available in the mitochondria. The fatty acids with numerous double bonds would have originally incorporated mature CL if the cells were in normal condition. Due to the dysfunction of CL remodeling, the unsaturated fatty acyl moieties were incorporated into PS and decreased the PS saturation (Figure 5D).

### 4.3. PG Supplementation

Symmetrical CL species can be found in animal cells and tissues [20]. PG(18:1)_2_ supplementation was incorporated into CL and drastically increased the level of mature CL(18:1)_4_ without the remodeling process in HAP1Δ*TAZ* cells. In the condition of remodeling dysfunction, PG(18:1)_2_ supplementation was an effective method to increase mature CLs. In HAP1Δ*TAZ* cells, most of the supplemented PG was efficiently used by other phospholipids, although the total concentration and the supplemented species of PG also elevated. The supplemented PG(18:1)_2_ exhibited an ideal fatty acyl chain combination for cellular use.

PG(18:1)_2_ supplementation did not increase the total concentration of CL but substantially changed the species of CL, including the symmetrical CL(18:1)_4_. The MLCL concentration considerably decreased, resulting in a reduction of MLCL/CL index for a healthy mitochondria. The morphology of the mitochondria had recovered from halfmoon shape to a regular oblong or oval shape. The inner membrane also recovered from the bubble like membrane to a cristae of layered inner membrane. The phospholipid compositions of the inner and outer membranes are crucial to maintain the morphology of mitochondria and the structure of mitochondrial cristae. We have observed up-regulation iPLA_2_β and iPLA_2_γ after *TAZ* knockout. Previous research has been shown that iPLA_2_β knockout can rescue the spermatogenesis, spermatid individualization, and MLCL/CL in a tafazzin-deficient *Drosophila* model of Barth syndrome [65]. Therefore, it is important to inhibit mitochondrial iPLA_2_(s) to prevent the hydrolysis of CL. In this study, the genes of iPLA_2_β and iPLA_2_γ were down-regulated upon PG supplementation, which caused the decreases of MLCL and lowered the ratio of MLCL/CL. Based on the elevation of the 18:1 moieties in the phospholipid, we could know that excessive supplemented PG(18:1)_2_ was either maintained as PG, or converted to PC and PE but not to PS (Figure 10). From the results, we can predict PS synthases, PSS1 and PSS2 could be both inhibited for PS production.

## 5. Conclusions

We have successfully established a Barth syndrome model by HAP1 cells to study the lipid compositions and mitochondrial morphology. Tafazzin knockout by CRISPER-Cas9 caused the decreased concentration level of CL and the accumulation of MLCL, which is resulted from the hydrolysis of CL by the phospholipases localized to mitochondria. The knockout also triggered the elevated level of PS concentration and reduced level of PC concentration. PG supplementation lowered the MLCL/CL ratio and the healthy mitochondrial morphology was observed under electron microscope. PG supplementation down-regulated two main mitochondrial phospholipases, iPLA_2_β and iPLA_2_γ, which suppressed the CL hydrolysis and lead to the decreased level of MLCL. Excess supplemented PG was shown remodeled to other phospholipids, such as PC and PE.

## Data Availability

The data available in this study are available on request from the corresponding author.

## Supplementary Materials

The following supporting information can be downloaded at: https://www.mdpi.com/article/10.3390/membranes12040383/s1, Table S1: Primers of RT-qPCR.
