# Supplementary material for: Phosphatidylglycerol Supplementation Alters Mitochondrial Morphology and Cardiolipin Composition
Source: Membranes (Basel). 2022 Mar 31;12(4):383. doi: 10.3390/membranes12040383 (PMC9028734; doi:10.3390/membranes12040383)
Supplement: Supplementary file 1 [file membranes-12-00383-s001.zip › membranes-1507790-supplementary.pdf]

membranes

MDPI

Article

Phosphatidylglycerol Supplementation Alters Mitochondrial Morphology and Cardiolipin Composition

I Chu [1,†], Ying-Chih Chen [1,†], Ruo-Yun Lai [1], Jui-Fen Chan [1], Ya-Hui Lee [1], Maria Balazova [2] and Yuan-Hao Howard Hsu [1,*]

[1] Department of Chemistry, Tunghai University, Taichung, Taiwan; joyce1024joyce1024@gmail.com (I.C.); michaelchen8900@gmail.com (Y.-C.C.); iz51034@yahoo.com.tw (R.-Y.L.); rivabox@hotmail.com (J.-F.C.)
[2] Institute of Animal Biochemistry and Genetics, Centre of Biosciences, Slovak Academy of Sciences, Slovakia; maria.balazova@savba.sk
* Correspondence: howardhsu@thu.edu.tw; Tel.: +886-4-23590121 (ext. 32230); FAX: +886-4-23590426
† These authors contributed equally to this work.
‡ Current address: Tunghai University, No. 1727, Sec. 4, Taiwan Boulevard, Xitun District, Taichung 40704, Taiwan.

Supplemental Table S1 Primers of RT-qPCR

GeneID	Accession Number	Forward OligoSequence 5'-3'	Reverse OligoSequence 5'-3'	Product Size (bp)	Temp (°C)	Intron Spanning
PGS1	NM_024419	GAACAGGAGCTGGTGGAC	CTAAGAGAATGGAGACCTTGAGAT	91	76	Yes
CRLS1	NM_001127458	AGCAGTCCAGTTAATCTT	TTATCACCTGAACAGTCTT	161	78	Yes
LCLAT1	NM_001002257	TGCTTGTCACAGAATCAT	CCAATAATGCCACAGGTA	191	79.2	Yes
PLA2G6	NM_001004426	GACCGAGATCCATGAGTA	ACAACGATGGAGAGTTTC	78	78	Yes
PNPLA8	NM_015723.4	GGGTGAGTATTGATAACAG	GAATAATTCTTTCCTTGACAG	152	-	Yes
GAPDH	NM_001289745	CACCGTCAAGGCTGAGAAC	GAGGGATCTCGCTCCTGG	75	77.8	Yes
